# The KBG syndrome: Case report

**DOI:** 10.1186/1757-1626-1-186

**Published:** 2008-09-26

**Authors:** Ilaria Morghen, Enrico Ferri

**Affiliations:** 1 Anesthesiology and Critical Care Department, S. Anna University Hospital, 203 C.so Giovecca, 44100 Ferrara, Italy

## Abstract

**Introduction:**

The KBG syndrome is a rare autosomal dominant condition, first described by Hermann *et al. *in 1975. Fundamental findings are: mild development delay, short stature, craniofacial dysmorphism and skeletal anomalies.

**Case presentation:**

A 32 years old woman, Caucasian race, weight 57 Kg, affected by KBG syndrome was sent to our clinics for preoperative anaesthesia evaluation. She was schedules for left ossicular reconstruction under general anaesthesia for bilateral hearing loss. A psycho-motor retardation was associated to morphological anomalies such as short neck, hyperlordosis without neck extension imparirment, craniofacial anomalies and dento-skeletal abnormalities. An echocardiography showed the presence of interatrial defect with left-to-right shunt. The patient was sent to a cardiac surgery centre.

**Conclusion:**

Perioperative evaluation of patients affected by KBG syndrome must take into consideration the management of difficult airways, due to the associated craniofacial dysmorphism. The possible presence of cardiac anomalies in the KBG syndrome is currently being evalueted. In this report the finding of cardiomegaly and congestion of the pulmonary circulation was attributed to presence of an interatrial defect with left-to-right shunt. The risk of cardiopulmonary failure led us to ask for a cardiac surgery consult. Perioperative management of these patients should be extremely accurate, even in the case of minor surgery, and should include also chest X-rays and echocardiography evaluation.

## Introduction

The KBG syndrome is a rare autosomal dominant condiction, first described by Hermann *et al. *in 1975. KBG are the initials of the first patient described whit this syndrome [1].

Fundamental findings are: mild developmental delay, short stature, craniofacial dysmorphism with brachycephaly, round face, hypertelorism, palpebral fissures in the mongoloid position, macrodontia of the maxillary permanent central incisors, hypodontia, short alveolar ridge, hearing loss for bilateral recurrent otitis media. Skeletal anomalies can include: cervical ribs, hip dysplasia, thoracolumbar scoliosis, anomalies of hands, femoral and vertebral bones [2-4].

We report a case of KBG syndrome being evaluated for ossicular reconstruction under general anesthesia, that was redirected to a cardiac surgery centre.

## Case presentation

A 32 years old woman, Caucasian race, weight 57 Kg, affected by KBG syndrome was sent to our clinics for a preoperative anesthesia evaluation. She was scheduled for left ossicular reconstruction under general anesthesia for bilateral hearing loss. The patient was born after a normal uncomplicated pregnancy.

Medical history: operation of congenital luxation of the hip to 19 years in spinal anesthesia.

Clinical examination showed a psycho-motor retardation associated to morphological anomalies, such as short stature, short neck, hypelordosis without neck extension impairment, mild hyperterolism, slight antimongoloid, extreme flattening of the face, arhinencephalic profile with right-convex deflection of the nasal septum, macrodontia of the upper central incisor, 1.1 and 2.1, hypodontia, anterior inverted bite (Mallampati class 3).

Thoracic examination revealed a 2/6 systolic cardiac murmur at the apex. Chest X-ray showed cardiomegaly, signes of congestion in the pulmonary circulation and increased hilar shapes. Since many years the patient had been reporting dispnoea with every airways inflammation process occurring in the past years.

Pre-operative evaluation with an echocardiogram showed the presence of biatrial enlargment, interatrial defect (IAD) with left-to-right shunt, right ventricle enlargment, estimated pulmonary artery pressure (PAP) 45–50 mmHg.

Electrocardiogram showed a right bundle branch block.

No left ventricle anomalies were found. Therefore the patient was sent to a cardiac surgery centre.

## Discussion

Perioperative evaluation of patients affected by KBG must take into consideration the management of difficult airways, due to the associated craniofacial dysmorphism. In this case, problems arose from the extreme flattnes of face, Angle class III malocclusion, short neck with hyperlordosis and macrodontia of the upper central incisors. High Mallampati score (Type 3) were assessed.

The possible presence of cardiac anomalies in the KBG syndrome is currently being evaluated. At present a single case of interventricular septum defect, two cases of bicuspid aortic valve and partial atrioventricular canal defect, one stenosis of the left pulmonary artery and a ventricular septal defect, has been reported [5-7].

Atrial septal defect occurs as an isolated anomaly in 5% to 10% of all congenital heart defects. It is more common in females than in males and can occur in any portion of the atrial septum. Right axis deviation and mild right ventricular hypertrophy or right bundle branch block are typical findings on electrocardiogram. Cardiomegaly with enlargment of the right atrium and right ventricle may be present. A 2-dimensional echocardiography is diagnostic [8].

The finding of cardiomegaly and congestion of the pulmonary circulation was attributed to presence of an IAD with a functional significance of the left-to-right shunt. The risk of cardiopulmonary failure led us to ask for a cardiac surgery consult. Upon agreement with the head and neck surgeon, we decided to postpone the ossiculoplasti intervention, this being not urgent for the patient, who carried an acoustic prothesis for bilateral mixed hearing loss since the age of 8. Although she had always been followed from a specialized centre for paediatric diseases, such a severe cardiac anomaly, undoubtably congenital and worsened during time, has been detected very late.

## Conclusion

Patients affected by KBG syndrome can present many challenges to the anaesthesiologist that performs the preoperative evaluation. This addresses the issue of considering the possible association of evolving cardiac anomalies when dealing with the KBG syndrome. Perioperative management of these patients should be extremely accurate, even in the case of minor surgery, and should include also chest X-rays and echocardiography evaluation.

## Abbreviations

IAD: interatrial defect; PAP: pulmonary artery pressure.

## Competing interests

The authors declare that they have no competing interests.

## Authors' contributions

MI has performed the preoperative evaluation, conceived the paper, searched the literature for other similar reports and review on the subject, drafted the manuscript. FE has been involved in the analysis of data and helped to draft the manuscript. All authors read and approved the final manuscript.

## Consent

Written consent was obtained from the patient for pubblication of this case report and any accompanying images. A copy of the written consent is avaible for review by the Editor-in-Chief of this journal.
